# A Young Woman Presenting With a Post-traumatic Tracheal Wound

**DOI:** 10.7759/cureus.39452

**Published:** 2023-05-24

**Authors:** Latifa Boukhidous, Badr Kharouaa, Sara Zarouki, Rachid Marouf, Afaf Thouil, Hatim Kouismi

**Affiliations:** 1 Department of Respiratory Diseases, Mohammed VI University Hospital, Faculty of Medicine and Pharmacy of Oujda, Mohammed First University, Oujda, MAR; 2 Department of Thoracic and Cardio-Vascular Surgery, Faculty of Medicine and Pharmacy of Oujda, Mohammed VI University Hospital, Mohammed First University, Oujda, MAR; 3 Department of Respiratory Diseases, Mohammed VI University Hospital, Oujda, MAR; 4 Laboratory of Research and Medical Sciences (LRSM), Faculty of Medicine and Pharmacy, Mohammed First University, Oujda, MAR

**Keywords:** subcutaneous emphysema, thoracic scanner, pneumomediastinum, bronchial fibroscopy, tracheobronchial ruptures

## Abstract

Tracheobronchial injury is a rare but potentially fatal occurrence, with most cases resulting from penetrating trauma or blunt and iatrogenic injury during medical procedures such as endotracheal intubation, bronchoscopy, or surgery. Early recognition of clinical symptoms can help stratify patient risk and guide management, though these symptoms are often non-specific. We report the case of a 42-year-old patient who presented with post-traumatic chest pain from a sharp object. Radiographic investigation revealed pneumomediastinum and significant subcutaneous emphysema, while bronchial fibroscopy confirmed a wound on the posterior surface of the trachea. The patient underwent surgery with an uneventful postoperative course. Follow-up radiographic evaluation three weeks later showed healing of the tracheal wound and good clinical improvement.

## Introduction

Tracheobronchial injuries are uncommon yet severe complications of chest trauma, encompassing all disruptions to the continuity of the tracheobronchial tree [1]. They are more frequently observed in cases of penetrating trauma than blunt trauma [2]. While history, clinical signs, and chest radiography are essential for initial evaluation, they are often nonspecific [2]. Therefore, healthcare providers must promptly initiate additional explorations, such as bronchial fibroscopy, which can confirm the diagnosis and guide therapy, thereby influencing prognosis. 

## Case presentation

**Figure 1:**
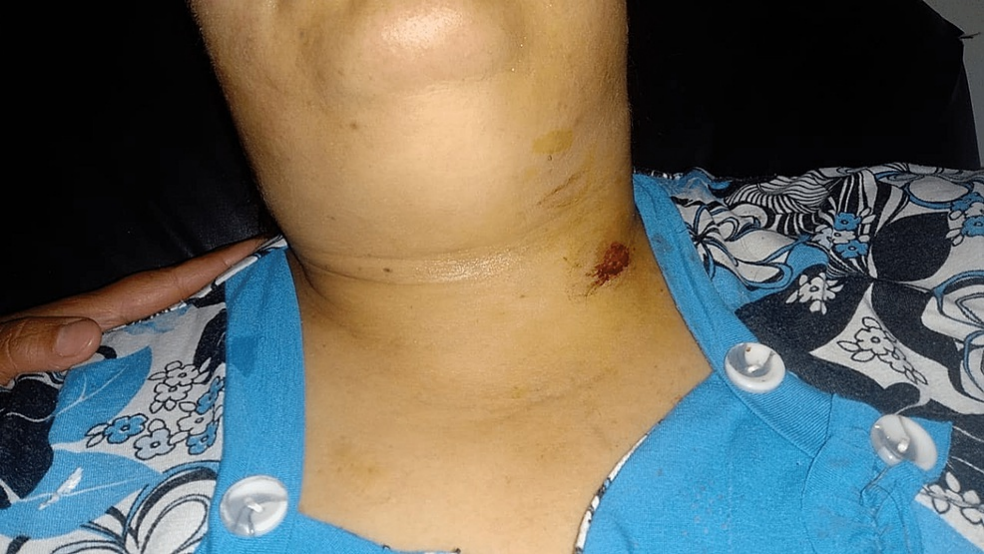
Image of the patient showing the location of the tracheal trauma.

A 42-year-old female patient presented to the emergency department with post-traumatic chest pain from a sharp object. Upon admission, the patient was alert and oriented with stable hemodynamic parameters and an arterial oxygen saturation of 91% on ambient air. The patient had no dysphonia and was afebrile with an overall preserved general condition. Cervicothoracic emphysema and a left punctiform cervical wound were observed on cervical examination (Figure 1). Pleuropulmonary examination revealed a normal morphology of the thorax and subcutaneous emphysema extending up to the face. Vocal vibrations were well-transmitted, and vesicular murmurs were well-perceived, without dullness or hyper-resonance. The remaining physical examination was unremarkable.

**Figure 2:**
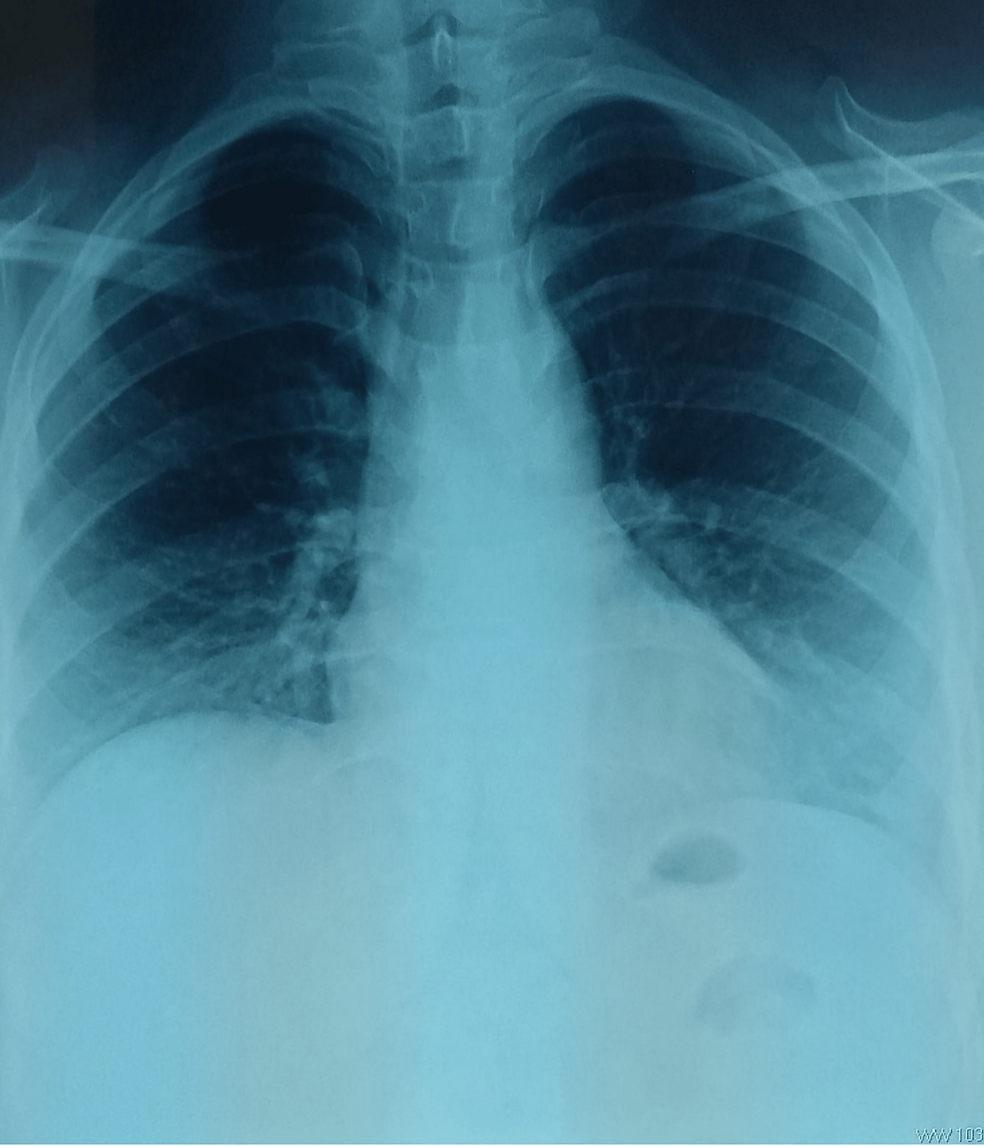
Chest x-ray showing parallel hyperclarity at the contours of the mediastinum more accentuated on the left side.

**Figure 3:**
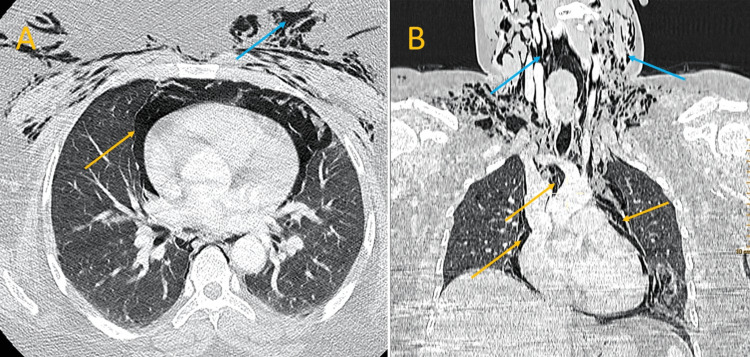
Injected thoracic CT scan in axial section (A), along with sagittal section (B), both in parenchymal window revealing extensive subcutaneous emphysema involving perivascular soft tissues and muscles of the cervical region (blue arrows), as well as massive pneumomediastinum (blue arrows) with esophageal filling

The chest radiograph demonstrated pneumomediastinum with a radiolucent line parallel to the heart borders (Figure 2). Computed tomography of the chest revealed significant subcutaneous emphysema extending to the perivascular soft tissues and muscles of the cervical region (Figure 3). The imaging also revealed an anterior tracheal wall defect at the level of the first thoracic vertebra, causing a massive pneumomediastinum and bilateral low-grade pneumothoraces. The esophageal lumen was filled with heterogeneous material, and a posterior parietal air bubble was observed without any contrast extravasation.

Emergency hospitalization was advised, and the patient was administered oxygen, antibiotics (amoxicillin + clavulanic acid 3 g/day), omeprazole (40 mg/day), and paracetamol (1 g/6 h) for symptomatic relief. Laboratory investigations showed a moderate inflammatory response, with leukocytosis of 10,440 cells/mm3 predominantly neutrophilic and elevated levels of C-reactive protein (CRP) at 80 mg/L.

**Figure 4:**
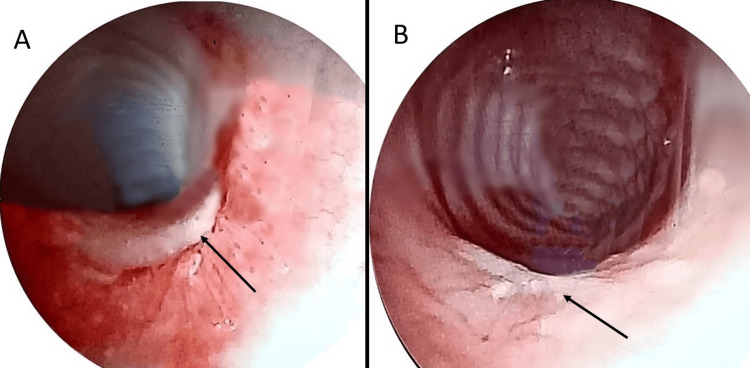
Endoscopic images of tracheal wound before surgery (A) and during a follow-up bronchoscopy after three weeks of surgery (B).

After the stabilization of the patient, bronchial fibroscopy identified a wound on the posterior surface of the trachea, located 2 cm distal to the vocal cords. There were no other visible abnormalities. A surgical exploration via left cervicotomy was performed, which included repair of the lateral-posterior tracheal perforation and a biopsy of a left cervical lymph node with drainage. A methylene blue test and Valsalva maneuver excluded an esophageal injury. The patient's postoperative recovery was uneventful, and a follow-up bronchoscopy performed three weeks later revealed complete healing of the tracheal wound and good clinical improvement. Figure 4 illustrates the endoscopic aspects of tracheal wound before and after surgery.

## Discussion

In accordance with the literature, clinical symptoms of post-traumatic pneumomediastinum are frequently characterized by subcutaneous cervical or thoracic emphysema and pneumothorax [3], which was evident in our patient. Other associated symptoms may include cough, dysphagia, or dysphonia [3]. Hamman’s sign, a crackling noise synchronous with heart sounds heard on auscultation of the precordial area, may also be present in approximately 50% of cases and is considered pathognomonic [3].

The first-line paraclinical examination for post-traumatic pneumomediastinum is a chest X-ray, which can identify several abnormalities [4]. A band of hyperclarity of the contours of the mediastinum parallel to the heart contours and descending thoracic aorta, and the presence of air in the soft tissues of the neck and thorax, indicating subcutaneous emphysema, are indicative of the condition. Levine’s sign, also known as the continuous diaphragmatic dome sign, is present if air is interposed between the heart and the diaphragm, and the flying thymus sign may also be visualized. Our patient presented with hyperclarity of the contours of the mediastinum parallel to the heart contours on chest X-ray [4].

CT scan is the gold standard for confirming the diagnosis of post-traumatic pneumomediastinum and may reveal tracheobronchial, oroesophageal, and bone lesions causing pneumomediastinum, as well as the presence of pneumothorax [3]. Depending on the patient's history and CT scan results, a CT scan with digestive opacification may be needed to search for esophageal perforation or a bronchial endoscopy to search for a tracheobronchial lesion. Bronchoscopy is the gold standard for searching for tracheobronchial wounds [5], although it may not be available in an emergency situation [6]. Finally, the management of post-traumatic pneumomediastinum is based on the underlying cause [3].

## Conclusions

The main interest of this work is to highlight the importance of bronchial fibroscopy in the diagnosis of tracheal wounds. The main clinical sign is cervical subcutaneous emphysema. In the end, the tomodensitometry with reconstructions must be carried out urgently in order to allow multidisciplinary therapeutic management.
